# The *Camino Verde* intervention in Nicaragua, 2004–2012

**DOI:** 10.1186/s12889-017-4299-3

**Published:** 2017-05-30

**Authors:** Jorge Arosteguí, Robert J. Ledogar, Josefina Coloma, Carlos Hernández-Alvarez, Harold Suazo-Laguna, Alvaro Cárcamo, Rosa María Reyes, Alejandro Belli, Neil Andersson, Eva Harris

**Affiliations:** 1 CIET Nicaragua, Managua, Nicaragua; 2 CIET International, New York, NY USA; 3 Centro de Investigación de Enfermedades Tropicales, Universidad Autónoma de Guerrero, Acapulco, Guerrero Mexico; 4 Department of Family Medicine, McGill University, Montreal, Canada; 5 Division of Infectious Diseases and Vaccinology, School of Public Health, University of California, Berkeley, CA USA

**Keywords:** Dengue, *Aedes aegypti*, Brigadista, Community mobilisation

## Abstract

**Abstract:**

*Camino Verde* (the Green Way) is an evidence-based community mobilisation tool for prevention of dengue and other mosquito-borne viral diseases. Its effectiveness was demonstrated in a cluster-randomised controlled trial conducted in 2010–2013 in Nicaragua and Mexico. The Nicaraguan arm of the trial was preceded, from 2004 to 2008, by a feasibility study that provided valuable lessons and trained facilitators for the trial itself. Here, guided by the Template for Intervention Description and Replication (TIDieR), we describe the Camino Verde intervention in Nicaragua, presenting its rationale, its time and location, activities, materials used, the main actors, modes of delivery, how it was tailored to encourage community engagement, modifications made from the feasibility study to the trial itself, and how fidelity to the process originally designed was maintained. We also present information on costs and discuss the place of this study within the literature on implementation science.

**Trial registration:**

ISRCTN27581154.

## Background: rationale, theory and goals

The dengue pandemic has continued to grow despite widespread use of temephos, and resistance to this pesticide is well documented. Though several studies have shown an impact of community interventions on vector control, no previous studies had shown an impact on dengue illness or serological evidence of infection. The Camino Verde project was the first to demonstrate, by way of serological evidence, a positive impact of pesticide-free community mobilisation on dengue virus infection in children and reported dengue illness at all ages. It also registered reductions in all *Aedes aegypti* control indices [1].

In Managua, Nicaragua, CIET initiated the *Camino Verde* (Green Way) approach to dengue prevention and control with a feasibility study from 2004 to 2008 [2]. The study outcomes -- lower entomological risk and dengue virus infection rates -- served as a basis for a much larger, two-country cluster randomised controlled trial in Mexico and Nicaragua in 2010–2013.

The importance of thorough feasibility studies to explore key issues and test activities before designing and launching a complex intervention is well recognised [3]. The trial validated the experience of the feasibility study, along with the ethical principles that guided it.

The feasibility study was the training ground for the two-country cluster randomised trial. People who had served as *brigadistas* in the feasibility phase became key personnel during the Nicaraguan arm of the trial, and members of the Nicaraguan team helped to train the field team for the Mexican arm of the trial as well.

The results of the Camino Verde trial have been presented elsewhere [1]. Here, guided by the Template for Intervention Description and Replication (TIDieR), we describe the Camino Verde intervention as it was conducted in Nicaragua [4].

## Where

The city of Managua, Nicaragua’s capital, is the centre of the country’s main financial, commercial and service activities. The urban area covers 150 km^2^, and the approximate population at the start of the trial was some 1.25 million, a fourth of the national total. Managua comprises seven administrative-political territories called districts, which cover some 541 low and lower-middle income neighbourhoods, 94 upscale residential enclaves and 21 rural neighbourhoods *(comarcas)* [5]*.* Low and lower-middle income neighbourhoods account for 89% of the city’s residents and comprised the population base for the selection of the 60 clusters that made up the trial’s measurement and intervention framework. The 11% of the population excluded from the trial selection lived in upscale residential neighbourhoods that have enough resources to meet their needs without requiring institutional assistance, and in suburban *comarcas* with more compromised security conditions.

Managua’s climate is tropical, warm and wet. The maximum and minimum altitudes are 600 m and 48 m above sea level, respectively. In the trial’s follow-up survey, we found that during the 2013 dry season, 43% of households stored water in barrels and had experienced irregular supply of piped water during the previous week, due to programmed service cuts, to a technical problem in the supply network, or to other operational problems [5].

## When and how much

The feasibility study was carried out between 2004 and 2008. The trial took place from mid-2010 to early 2013. Both phases included baseline and follow-up surveys and in each case, these surveys involved the collection of saliva samples from children between 3 and 9 years of age in two stages, before and after the dengue season, in order to detect recent dengue virus infection. The trial intervention itself occurred between July 2011 and December 2012.

During the feasibility study, we conducted four survey cycles with a total of eight measurements among 3200 households from 30 neighbourhoods (10 intervention, 20 control). During the trial, we carried out two cycles that included three entomological surveys (two in the dry season and one during the rainy season) among 8100 households from 60 neighbourhoods (30 intervention, 30 control). We assessed dengue virus transmission risk by collecting saliva samples from 2900 children aged 3–9 years during the feasibility study and from 4600 children during the trial.

Both phases of Camino Verde involved close collaboration with the Ministry of Health’s National Diagnostic and Reference Center (CNDR), which directly participated in producing serological and entomological evidence. The entomological portion of these surveys was directed by two supervisors, one of whom either had responsibilities in the vector control programme of the local healthcare system (known according to its Spanish acronym as the SILAIS) or was a CNDR entomologist. The entomological inspection applied the protocol and techniques standardised by the Nicaraguan vector control programme for collecting, transporting, conserving, identifying, counting and classifying immature *Ae. aegypti* specimens at different stages of development. The immunological part consisted of measuring anti-dengue virus antibodies by Enzyme-linked Immunosorbent Assay (ELISA) in paired saliva samples collected before and after the dengue season to detect an increase indicative of dengue virus infection during that period.

## Materials

The principal material used in Camino Verde was evidence. The evidence was biological, entomological, epidemiological, and economic. The biological evidence concerned the lifecycle of the *Aedes aegypti* mosquito and the development stages of the aquatic immature forms. The entomological evidence was of two kinds: a) aggregate numbers and percentages of mosquito larvae and pupae derived from systematic inspections of household and community water receptacles and b) visual demonstration of the presence of these larvae and pupae to householders on their own premises. The epidemiological evidence came from the baseline study and concerned risks from failure to protect against dengue and likelihood of protection from various actions that households and communities could take to minimize those risks. The economic evidence was a) cost data on dengue and dengue control gathered in the baseline surveys and b) reflection by each household on the costs they incur from seeking treatment for dengue illness and from purchases of anti-mosquito chemicals and devices.

Among the tools used by brigades during household visits were larval nets, plastic pans, plastic pipettes, magnifying glasses, and flashlights. A particularly important tool was a laminated graphic showing the mosquito vector’s life cycle on one side, with alternative control measures and the advantages and disadvantages of each option on the other. Linking the adult *Aedes aegypti* mosquito with its immature forms made all the difference in people’s understanding of the situation and stimulated decisions about the most convenient way to avoid the mosquito, be it "not providing it with a home" or “preventing it from taking flight,” using the physical evidence found in the households themselves.

The materials used in collective actions such as clean-up campaigns, parades and festivals were provided by the community members themselves.

## Procedures

The intervention was preceded by a baseline survey among 8100 households from 60 neighbourhoods (30 intervention, 30 control). Paired saliva samples were collected from 4600 children in mid-2010 at the beginning of the dengue season and at the end of it in early 2011, in order to detect recent dengue infection. This process was repeated during the follow-up survey in mid-2012 and early 2013. In January 2011 (and again in 2013) we returned to all households in both intervention and control communities to inform parents of the results from the analysis of the saliva samples for each child who had provided two samples along with recommendations for what action, if any, they should take.

The intervention itself was based on a process called Socialisation of Evidence for Participatory Action (SEPA), a communication strategy that bolsters direct community participation by fostering evidence-based dialogue and looking for solutions on the communities’ own terms [6]. In Nicaragua, the SEPA methodology underwent constant enrichment from the project’s protagonists –households, community collectives, neighbourhood leadership and participating institutions – and it was grounded in knowledge gained and systematized from the experiences of the feasibility study.

In May and June of 2011, as a way of gaining access to the neighbourhoods and getting to know the community residents, CIET staff invited community leaders from the 30 intervention neighbourhoods to participate in discussions about the costs incurred by neighbourhood families in relation to dengue as revealed by the baseline survey. The topic of household budget decisions provided a good entry point for researchers to engage with communities, especially when the evidence showed that current expenditures were providing a poor return. People became motivated not only to search for ways to reduce their costs but also to question the current response to the mosquito problem. This in turn helped people create conditions favourable for community mobilisation for change. The results of this activity are reported in a companion article [7].

The dialogue did not attempt to impose externally pre-defined behaviours, which implies a biased relationship of superiority on the part of those who define the behaviour. The informed dialogue was rather conceived as a horizontal relationship where evidence facilitated the conversation about the dengue problem and possible options, and its ultimate effect was motivation for responsible action.

After the focus groups, beginning in June 2011, the community collectives called SEPA brigades took shape. These were conceived as groups of diverse residents who voluntarily organized to prevent dengue in their neighbourhoods. These groups were more than just ad hoc groups created only for the project; rather, they emerged from within the community to address a community problem. They did this by acting as a link between the population and community leaders and by conducting household “accompaniment visits” and collective actions in the neighbourhood. Members of the SEPA brigades were called *brigadistas* (See below under Actors).

### Household “accompaniment visits”

Household visits were the occasion for informed dialogue with household heads and members. Dialogue was complemented by evidence and emphasized finding solutions suggested by the household members themselves. This implied reviewing what was already being done in the household and, where necessary, looking for alternative solutions based on the evidence from each of the *Aedes aegypti* breeding sites on the premises. The dialogue included ways of dealing with the evidence using the household’s own resources, without preconceived recipes or solutions.

At first, the visits covered only the approximately130 households in each neighbourhood that had been included in the baseline survey. Each team of two *brigadistas* had a specific neighbourhood sector or block assigned to it. As time went on and human resources allowed*, brigadistas* visited additional households in the same residential sector. The trend was to expand the range of visits in order to cover the whole neighbourhood, as the additional households were increasingly considered to be part of the community organization’s everyday efforts. In principle, each household received a weekly visit from the same team, although the regularity depended above all on the household’s entomological condition and vector control capacity. The brigades were soon able to judge from a short visit how well a household was managing its water receptacles, especially households with children who could more easily identify the immature forms of the mosquito. As time went on, brigades were visiting some households more than once a week and others only occasionally.

The moment of knocking on the household door and the initial interaction with residents became a critical point in the relationship between brigades and community members, as it condensed in a single occasion the issues of consent, trust, and confidentiality. Permission to enter the home imposed upon the *brigadistas* a commitment not to make public what people considered private when discussed in their households.

Once *brigadistas* were allowed inside the dwelling, the joint inspection of water receptacles and conversation about new experiences or changes observed since the last visit constituted moments of dialogue in which no one’s knowledge was considered necessarily superior to another’s; participants simply exchanged interpretations and, with the help of some simple educational tools, identified feasible, actionable alternatives. In this manner, the brigade was perceived as a part of the community with recognition of its voluntary character and its service mission.

### Collective actions

Aside from household visits, the purpose of SEPA brigades was to build collective responsibility to tackle common problems beyond the households, whether in the street, the sector or the whole neighbourhood. Their work routine involved participating in community actions jointly with neighbourhood leaders, using evidence whenever possible.

A first level of collective responsibility focused on making it clear that having *Ae. aegypti* breeding sites in the household created a risk of dengue for neighbours. This fostered awareness of how each household could affect others, and how other households could affect one’s own. Starting from this awareness, neighbours were stimulated to talk with one another about how to control breeding sites and about mutual responsibility and even solidarity when a household did not have the necessary resources to carry out the appropriate tasks (e.g., households with only elderly residents).

More broadly, collective activities included, for example, participating in community clean-up sessions, educational activities during community festivals, and reforestation activities; repairing road infrastructure using old tires and scrap materials, thus converting such materials from potential mosquito breeding sites into useful resources; conducting educational and entomological control activities in schools and workplaces, etc. Communities carried out these dengue prevention activities in coordination with institutions such as the Managua city hall, the Ministry of Health, the Ministry of Education and the Ministry of the Environment and Natural Resources, or used their own resources to meet the neighbourhood’s demands in their streets, parks or schools.

The progress of the SEPA process allowed for increasingly closer ties between the brigades and community leaders. Thus, just a few months after the start of the project, the SEPA brigades had already become an important part of the community dynamics and structures in several neighbourhoods. Further details, including photographs, of these activities can be found at: http://caminoverde.ciet.org/en/nicaragua/activities/.

### Meetings with public institutions

Other events aimed at strengthening relationships between communities and the Ministry of Health around the subject of mosquito prevention. For example, community leaders and SEPA brigades from three neighbourhoods accompanied representatives of Managua’s local health authority in launching the first Day to Combat Dengue in 2012. At this event, *brigadistas* and health authorities accompanied community residents in entomological inspections of their households, showing them the larvae and pupae they found. The participants also discussed the burden on household economies from the costs of dengue illness or insecticide use.

Another event that linked the SEPA process with the Ministry of Health took place in the CIET office. The event was a meeting between community leaders from 14 intervention neighbourhoods and staff from the government’s Vector-borne Disease Control Program. The meeting involved an exchange of epidemiological and entomological information, and both Ministry of Health personnel and community leaders showed each other how they visualized their challenges and responsibilities. CIET presented the entomological trends identified by the peer monitoring work (see below), while the government programme director presented the occurrence of registered cases and the results of an entomological survey carried out by the government a few months before the meeting.

The exchange of evidence fostered a dialogue aimed at joint actions by the Ministry of Health and the communities in two sensitive areas: information exchange and joint action on the *Aedes aegypti* mosquito’s breeding sites that were beyond the households’ control, which were called “critical points”, and included ditches, empty lots, scrapyards, recycling dumps, tire repair shops, workshops, factories, or any other place that was beyond the community’s capacity to control.

## Actors

### Brigadistas

The brigades were made up of community leaders who had taken part in the focus groups and other community members invited by the leaders. Their members became known as *brigadistas*. The only requirement for participation was the motivation to prevent dengue and the voluntary decision to devote some time each week to visiting households or carrying out some neighbourhood action. Volunteers were motivated to join the brigades by the desire to participate in the collective experience and to do something for their communities.

As *brigadistas* worked, they learned and developed an ethic of respect, above all respect for privacy, but also respect for each household’s diversity and self-determination. There was a total of 29 brigades (one per intervention neighbourhood), with members of different ages and genders. By January 2012, there were 474 active *brigadistas* in 29 collectives, with an average of 16 per brigade and a range of 7–25 members. Thirty-seven percent of *brigadistas* were children (20% boys and 17% girls); 31% were teenagers (12% male and 19% female); and 31% were adults (8% men and 24% women). In some brigades, members were predominantly children; in others, they were predominantly teenagers; and in others they were predominantly adult housewives. The rest of the brigades had mixed memberships. The number of *brigadistas* varied constantly due to the arrival of new members, or the temporary or permanent withdrawal of others.

Within each brigade, *brigadistas* chose a coordinator to serve for a specified period. She or he could be re-elected as many times as the brigade chose, or removed from the post if the brigade so decided. In January 2012, 21 of the coordinators were women and 8 were men, and 16 brigades were directly coordinated by a delegate from the official neighbourhood leadership. The brigades met with varying regularity, usually once a week, in order to discuss their work plans. Decisions were preferably made by consensus. The leadership kept track of these decisions by way of regular meetings with and among the brigades and also through the monthly accounts received from the brigades as discussed below under the heading of Costs. Throughout most of the trial, the brigades trained new members themselves.

### Facilitators

In addition to the research team, CIET deployed a group of external community facilitators, whose presence as outsiders to the intervention neighbourhoods was justified insofar as they brought new knowledge into the community and supported the systematization of the communities’ own knowledge and experiences. Brigade members from intervention neighbourhoods in the feasibility phase became facilitators during the trial.

Several of the neighbourhood leaders who had begun to “develop the SEPA way” during the feasibility stage continued on as leaders in their own communities, while 19 (17 women and 2 men) joined the project management team of the trial as facilitators, with the aim of having a direct and temporary presence in the intervention neighbourhoods. The facilitators’ first task was to assess the dynamics of their assigned neighbourhoods, their people, and the neighbourhood’s key mosquito-generating points. They later took care of returning baseline results back to the households, coordinating focus groups and, together with community leadership, organizing the brigades and providing the necessary tools and work supplies for household visits. Later elements of their role as facilitators came when they promoted the decentralization of the brigades’ work, accountability, territorial expansion of the work, and neighbourhood peer monitoring.

At the beginning of the project, the relationships established between CIET facilitators and communities were slightly hierarchical. But facilitators had learned in the feasibility stage the need for a progressive shift on the part of leaders toward respectful facilitation that stimulated independent development opportunities in a context of shared responsibility. During the trial itself, CIET facilitators progressively withdrew from the communities. The fact that CIET facilitators came from other neighbourhoods to collaborate in endogenous communication processes in a neighbourhood that was not their own was both a challenge and an important point in the development of Camino Verde, specifically in preserving the ethic of respect that had to be present throughout the entire process with regard to local leadership and the brigades’ bond with households and the population.

The work of the facilitators was crucial to the SEPA process, including their influence on the brigades, the growing participation of community leaders and ultimately the preparation for their own withdrawal from the neighbourhoods. This exit process was progressive and depended on the brigades acquiring autonomy and self-sufficiency in their organization skills.

## Modes of delivery

In the case of the household visits, the mode of delivery was face-to-face with the household head and other household members wherever possible. For collective actions, the initial interface was also face-to-face between *brigadista* and community leadership so that the actions themselves were led by the latter.

### The SEPA blog

A few months after the start of the trial, with the aim of creating a space for communication among the intervention neighbourhoods, we launched an electronic platform where community *brigadistas*, residents and leaders could give testimonies of their own experiences, share anecdotes, enjoy photos, access their own data and work tools, monitor the product of their own work, and ask, comment and learn about what was happening in other neighbourhoods. This SEPA blog (http://sepa-nic.blogspot.com) provided "a space that makes visible committed individuals who anonymously offer their solidarity, receiving in exchange their own satisfaction and social recognition" [8].

A few weeks before the launch of the blog, 39 *brigadistas* from 23 neighbourhoods received training on how to use the software. The activity consisted of a demonstration complemented by practical activities with different features of the platform – calendar, image gallery, videos, data, tools – and how to comment. At the *brigadistas’* own request, the group was called the “SEPA cybernetic group”. This group was responsible for motivating and facilitating blog access by brigades and community leaders in their neighbourhoods, providing technical support for access, browsing and commenting, and contacting the person charged with the managing the blog at the CIET office. Only ten group members had direct access to the internet in their own homes (10/39), while the others had to visit internet cafés in their neighbourhoods or the home of a relative, friend or neighbour. The group agreed that each SEPA neighbourhood would have its own blog account, for security reasons and to strengthen the neighbourhood’s sense of identity. Each neighbourhood had its own password, as access to the tool was restricted to the intervention neighbourhoods throughout the duration of the trial.

## Tailoring

The Camino Verde intervention was tailored at several levels. At country level, it was tailored to the different political, cultural and socioeconomic conditions of Mexico’s Guerrero state and those of Nicaragua’s capital city, Managua. Appendix 2 to the main report on the trial results presents the main similarities and differences in how the intervention was implemented in the two countries [1].

Within Managua, researchers learned from the feasibility study how different the distinct households, sectors and neighbourhoods were from one another, and, because of this, how different were their interpretations of, and capacities to deal with, the problems they encountered. The dialogue between researcher and communities had to be adapted to the particular circumstances, obliging the former to move beyond academic discourse, preconceived ideas and uniform recipes and, instead of arriving to teach, to approach the neighbourhoods ready to learn.

By discussing their own data, brigades systematized their understanding of the different territories in which they worked (blocks, streets, households). In this way, brigade activities were not programmed with a predefined norm, but with recognition of the singularities of each neighbourhood and each block, resulting in household visit plans with specific priorities, contents and frequencies.

## Modifications

The intervention, understood as a process rather than as a fixed set of procedures, was not substantially modified from the inception of the feasibility study to the end of the trial, but it was refined and tailored, as mentioned above, during the course of that period.

An example of how robust the process was can be seen in its application to the feasibility and the trial phases of *Camino Verde*. The two phases developed in markedly different contexts as regards the relation between citizens and state institutions. The feasibility study took place during a political period in Nicaragua when the community voice was not officially promoted, there was minimal community mobilisation, and the presence of government institutions in the neighbourhoods was hardly noticeable. The 2010–2013 trial, on the other hand, took place at a time of major political changes when there was an increased presence of government programmes promoting community organizations, with local political leaders implementing social programmes, and an increased awareness among residents of the government’s vector control programme.

The different political and institutional contexts in which the feasibility study and the Camino Verde trial were developed in Nicaragua suggests that the Camino Verde protocol could be replicable in dissimilar socio-political contexts, as long as the process focuses on community residents, whose own motivations to participate and mobilise are grounded in their understanding of their own needs. This conclusion is further strengthened by the experience in the Mexican arm of the trial, conducted in the southern state of Guerrero, where community structures and leadership are more heterogeneous.

An example of how the process was refined over time can be seen in the evolution of the educational messages. At the beginning of the feasibility phase, we thought that most people were already aware of the connection between the adult mosquito’s existence and its immature forms, but this was not the case. Sharing this knowledge thus became a key element in the communication process.

## Fidelity to the intervention design

Three types of activity helped to assure fidelity to the intervention, although each of them served other purposes as well.

### Neighbourhood peer-monitoring

Aside from the work in their own neighbourhoods, brigades also engaged in formal peer monitoring and evaluation, with a brigade from one neighbourhood monitoring the work of its counterpart in another neighbourhood. In addition to assuring fidelity to the intervention design, these activities helped to strengthen *Aedes aegypti* prevention, broadened and strengthened the bonds among *brigadistas*, and created new learning experiences.

*Brigadistas* and community leaders from one neighbourhood requested permission from another neighbourhood to be allowed to pay a formal visit and enter that neighbourhood with their work tools, in order to perform measurements and to share the results. The visiting teams usually arrived in the neighbourhood in the morning and inspected households following a protocol that included collecting all the immature forms of *Ae. aegypti* they found. They also gathered household opinions about the neighbourhood brigade’s work, along with specific recommendations. The visits covered households located within the measurement cluster. The time available to the brigades, the number of visiting brigadistas, and the duration of the visits determined the number of visits.

At the end of each neighbourhood peer visit, the visiting team processed the results on site and shared the evidence with the host brigade and community leaders. They submitted collected larvae and pupae in clear plastic bags with labels identifying the source households. They shared the answers to the questionnaire and the visited households’ recommendations, in case there were any questions. Afterwards, the host brigade discussed the visiting brigade’s findings, comparing them with results from previous visits. At a later date, the roles were reversed: the host brigade became the visitor and the visitors, the hosts.

There were three cycles of peer monitoring visits between neighbourhoods. The first took place in the last week of November 2011, the second during the first week of January 2012, and the third in May 2012. Each peer monitoring cycle covered between 3500 and 4000 households.

The sense of neighbourhood responsibility for the outcome of the visits stimulated the process. The host *brigadistas* and community leaders were anxious to receive the entomological results concerning their own areas, giving them an opportunity to validate changes since previous measurements. In some neighbourhoods, larval and pupal positivity decreased. In others, no change was noted, while in others positivity increased. In any of these situations, the new evidence was cause for reflection on factors that could explain the outcomes at the household, neighbourhood and/or sector levels, and efforts to reach a consensus on options to improve the work. Also, neighbourhood peer monitoring broadened the perspectives of the brigades as they recognized that other situations differed from their own, and developed capacities for interpretation and dialogue reaching beyond their own territories.

### Activities among neighbourhood brigades

As part of the intervention, in addition to the peer monitoring activities, brigadistas and community leaders from different neighbourhoods took part in joint activities. Brigadistas and community leaders from one neighbourhood hosted these events, and teams from other neighbourhoods attended. These activities sought to create spaces for bonding and exchanging knowledge and experience among brigades and community leaders. These exchanges centred on household visits that took place in the host neighbourhood, identifying strengths and weaknesses. These gatherings also involved group integration activities, such as sporting events, contests and *piñatas* among other entertainment. The host community leadership coordinated the events, with CIET providing some educational materials and lending such items as banners and megaphones. Visits were usually returned at a later date.

The first of these events took place in April 2011 in the *La Primavera* neighbourhood, with some 90 *brigadistas* visiting from 6 neighbouring *barrios*. A second encounter took place in *Colonia 14 de Septiembre*, which was visited by the *Villa Libertad* and *Manuel Fernández* brigades. During the 2011 summer holidays, a series of meetings and events provided an opportunity for brigades to share in community activities and entertainment during periods of extreme heat in Managua.

### Events including all neighbourhoods

During the pilot stage and the trial, we organized four events involving all the intervention neighbourhoods. Three of these took place during the pilot stage and the fourth event took place during the trial. Some 600 delegates from the neighbourhoods, both brigadistas and community leaders, attended the last event, as well as national and international collaborators and funders. The event included presentation of results by the brigades, socio-dramas about the mosquito life-cycle and dengue, music and traditional dances. Overall the delegates and participants demonstrated a sense of pride and ownership of Camino Verde that strengthened participants’ sense of identity and belonging to a social movement with novel proposals to address the dengue problem. The SEPA blog gives details of the event.

## COSTS

The Camino Verde intervention in Nicaragua, though animated by volunteer brigades, was not free of costs. Monetary costs included part of the salaries of the research team which, in addition to its measurement activities, devoted a good deal of time to managing the intervention itself. There were payments to facilitators who, although they gradually withdrew from active presence in the communities, were indispensable in launching and guiding the early development of the brigades. And there were logistical and administrative costs as well. These costs will be detailed in an as yet unpublished report on the costs of Camino Verde by a health economist.

As for the brigades, individual brigadistas were all volunteers who received no individual payment. There was, however, a monthly fund averaging about US$55 provided to each brigade to cover the costs of supplies, transportation, communication and refreshments for meetings and collective actions. This fund was a contribution to sustain the brigades’ collective effort and to stimulate the development of their capacity for planning based on entomological results. At no point was the fund intended for individual use. In a few cases when this did happen, the brigades themselves determined the means for correcting the situation and sanctioning the person involved. While these resources were initially provided externally, the aim was for communities to progressively mobilise or generate their own resources as they reached greater levels of autonomy and sustainability. From the beginning, the amount provided to each community varied according to its perceived capacity to conduct the intervention using its own resources, and for some brigades the payment was suspended completely three or four months prior to the end of the intervention because they no longer needed it.

The brigade coordinator was accountable for the funds, both internally within the brigade and by providing accounting information to the CIET office, where officers registered it in the accounting system. Detailed financial accounting provided evidence of the brigade’s performance and allowed planning of the next work period in a transparent, collective way. The brigade coordinator acted as the link between the CIET office and the accounting system.

## Discussion

A July 2009 editorial by four members of the editorial board of the BMC journal *Implementation Science* observed that complex behaviour-change interventions are not well described, making it difficult to replicate them and limiting the possibilities for subsequent interventions to be successful [9]. Much of the early work in implementation science was written from clinical and institutional perspectives [10–12]. The authors of the editorial assumed that interventions are based on detailed manuals and urge that these be made public for use in replicating the intervention. They use the example of the “active ingredient” in pharmacology and cite a list of 137 separately defined techniques for behavioural change intervention [13].

Since 2004, on the other hand, Hawe and colleagues had been arguing that, in complex interventions, the function and process of the intervention should be standardized, rather than the components themselves, thus allowing the form to be tailored to local conditions [14].

The Camino Verde intervention in Nicaragua was designed from the latter perspective. The process standardised throughout the intervention, called SEPA, is the sharing (socialisation) of evidence with community residents and leaders – largely evidence of the mosquito larvae and pupae growing in their own water receptacles -- in ways that elicit household and community action to prevent the spread of dengue virus (and now chikungunya and Zika virus as well).

The experience of both the feasibility study and the intervention convinced the research team that evidence grounded in the specific reality of each household and community has greater potential for mobilisation than any didactic material proposing recipes that are equally applicable for everyone. Researchers also became convinced that evidence-based communication among households, volunteer groups and community leaders helps to increase neighbourhood solidarity and serves as a wellspring of new leadership.

We also found that monitoring and evaluation activities carried out by the communities themselves, along with the interpretation of changes brought on by their own efforts, were forces for community empowerment. Evaluations by peer groups and other external agents can complement communities’ own efforts.

The quality of external agents can be measured by their ability to shift from a role of leadership to one of respectful facilitation that stimulates opportunities for shared responsibility and autonomous development by the community. NGOs, academic institutions or other external organizations involved in similar interventions might learn from this experience and aim also for progressive withdrawal and eventual exit from the communities.

Recent unpublished measurements suggest sustainability of domestic and community knowledge and attitudes two years after the conclusion of the Camino Verde trial. Nevertheless, we need to develop more precise and sensitive sustainability indicators. At the household level, the elimination of breeding sites needs to become part of everyday household cleaning chores, along with the use of sustainable tools and lower dependency on chemical products. Sustainability of collective actions requires identifying community and institutional responsibility for cleaning and controlling breeding sites in communal areas, and continued prioritisation of mosquito control by community leaders and community volunteers.

## Conclusion

This article is not a manual for step-by-step application in other circumstances. It is a description of how the SEPA process was implemented under the particular conditions of one city in one country during the years 2010–2013. We believe, of course, that its content may be useful to others interested in applying the same process, or a similar one, to their particular circumstances, but only if they strive to adapt the process to those circumstances.

## Abbreviations

CNDR: *Centro Nacional de Diagnóstico y Referencia* (National Centre for Diagnostics and Reference)
SEPA: Socialisation of Evidence for Participatory Action
